# O Jejum Intermitente Associado a Dieta Low-Carb pode Prevenir Doenças Cardiovasculares em Pré-Diabéticos?

**DOI:** 10.36660/abc.20230182

**Published:** 2023-04-18

**Authors:** Luciana Nicolau Aranha

**Affiliations:** 1 Programa de Pós–Graduação em Medicina Universidade Federal do Rio de Janeiro Rio de Janeiro RJ Brasil Programa de Pós–Graduação em Medicina (Cardiologia) da Universidade Federal do Rio de Janeiro, Rio de Janeiro, RJ – Brasil

**Keywords:** Estado Pré-Diabético, Jejum, Dieta Pobre em Carboidratos, Estilo de Vida, Atividade Física, Exercício, Restrição Calórica

Pré-diabetes é o termo utilizado para descrever indivíduos que apresentam o metabolismo da glicose alterado, no entanto não atendem aos critérios diagnósticos de diabetes.^1^Pessoas com pré-diabetes têm um risco aumentando de diabetes mellitus tipo 2 (DMT2), doença cardiovascular (DCV) e mortalidade por todas as causas.^1 , 2^

A modificação no estilo de vida, incluindo dieta e prática regular de atividade física tem se mostrado efetiva na prevenção de diabetes e DCV.^3^Para promover perda de peso e controle glicêmico, algumas abordagens nutricionais têm sido utilizadas. O jejum intermitente é uma prática alimentar que envolve a restrição do consumo de calorias durante um período controlado, seguido do consumo calórico *ad libitum* .^4^ A dieta restrita em carboidratos, conhecida como *low-carb* é caracterizada pela ingestão de carboidratos abaixo do limite inferior da faixa aceitável de distribuição de macronutrientes para adultos saudáveis (ou seja, < 45% da ingestão de energia).^5^

Nesta edição dos Arquivos Brasileiros de Cardiologia foi publicado um estudo para analisar os benefícios do jejum intermitente associado a dieta *low-carb* em resultados micro e macrovasculares em pré-diabéticos.^6^ O estudo acompanhou por 2 anos pacientes pré-diabéticos que foram randomizados em 2 grupos: grupo I que realizou jejum intermitente (16 h - 3-4 dias por semana) acompanhado da ingestão de baixo teor de carboidratos (<26% da ingestão total de energia) e grupo II com ingestão calórica *ad libitum* . No final do acompanhamento os autores observaram menor peso corporal, índice de massa corporal, circunferência da cintura, percentual de gordura corporal, hemoglobina glicada e menor incidência de progressão para diabetes no grupo I. O grupo II apresentou aumento na incidência de complicações micro e macrovasculares na forma de retinopatia, neuropatia e angina instável.^6^

De acordo com esses resultados, a combinação de jejum intermitente com dieta *low-carb* pode ser capaz de prevenir DMT2 e DCV em pré-diabéticos.^6^ Outros estudos que combinaram estas estratégias alimentares também observaram resultados semelhantes.^7 , 8^ Apesar dos achados promissores, faltam evidências que comprovem a eficácia e segurança da utilização do jejum intermitente associado a dieta low-carb em pré-diabéticos, o que limita a generalização dos resultados. É importante enfatizar que estas práticas alimentares podem não ser adequadas para todos os indivíduos, sendo fundamental o acompanhamento por um profissional de saúde, que deverá adequar a dieta ao perfil de cada paciente, considerando os hábitos alimentares, o estado nutricional e as condições de saúde.

As diretrizes têm enfatizado para prevenção de diabetes e DCV em pré-diabéticos a importância de um padrão alimentar saudável, contendo vegetais, frutas, legumes e grãos integrais, bem como a escolha por fontes saudáveis de proteína e por alimentos in natura e minimamente processados, logo, vários padrões alimentares podem ser considerados Além disso, recomenda-se individualizar os planos alimentares com a distribuição de macronutrientes mais consistente com a preferência pessoal e a ingestão habitual para aumentar a probabilidade de manutenção a longo prazo.^9^
