# Supplementary material for: O Jejum Intermitente Associado a Dieta Low-Carb pode Prevenir Doenças Cardiovasculares em Pré-Diabéticos?
Source: Arq Bras Cardiol. 2023 Apr 18;120(4):e20230182. [Article in Portuguese] doi: 10.36660/abc.20230182 (PMC10263434; doi:10.36660/abc.20230182)
Supplement: Translation

# Translation: Can Intermittent Fasting Associated with a Low-Carb Diet Prevent Cardiovascular Disease in Pre-Diabetics?

**Authors:** Luciana Nicolau Aranha

**Affiliations:** 1 Brasil Programa de Pós–Graduação em Medicina (Cardiologia) da Universidade Federal do Rio de Janeiro, Rio de Janeiro, RJ – Brasil

**Keywords:** Prediabetic State, Fasting, Diet Low-Carbohydrate, Life Style, Physical Activity, Exercise, Caloric Restriction

Pre-diabetes is the term used to describe individuals who have altered glucose metabolism but do not meet the diagnostic criteria for diabetes.^1^People with pre-diabetes have an increased risk of type 2 diabetes mellitus (DM2), cardiovascular disease (CVD), and all-cause mortality.^1 , 2^

Lifestyle changes, including diet and regular physical activity, are effective in preventing diabetes and CVD.^3^Some nutritional approaches have been used to promote weight loss and glycemic control. Intermittent fasting is a dietary practice restricting calorie consumption during a controlled period, followed by ad libitum caloric consumption.^4^A carbohydrate-restricted diet, known as low-carb, is characterized by carbohydrate intake below the lower limit of an acceptable macronutrient distribution range for healthy adults (i.e., < 45% of energy intake).^5^

A study was published in the *Arquivos Brasileiros de Cardiologia* to analyze the benefits of intermittent fasting associated with a low-carb diet in micro and macrovascular results in pre-diabetics.^6^The study followed for 2 years pre-diabetic patients who were randomized into 2 groups: group I, who performed intermittent fasting (16 h - 3-4 days a week) followed by low carbohydrate intake (<26% of total energy), and group II with ad libitum caloric intake. At the end of the follow-up, the authors observed lower body weight, body mass index, waist circumference, percentage of body fat, glycated hemoglobin, and a lower incidence of progression to diabetes in group I. Group II showed increased micro and macrovascular complications such as retinopathy, neuropathy, and unstable angina.^6^

According to these results, combining intermittent fasting with a low-carb diet may prevent T2DM and CVD in pre-diabetics.^6^Other studies that combined these dietary strategies also observed similar results.^7 , 8^Despite the promising findings, there is a lack of evidence proving the effectiveness and safety of using intermittent fasting associated with a low-carb diet to reduce cardiovascular risk in pre-diabetics, which limits the generalization of the results. It is important to emphasize that these dietary practices may not be appropriate for all individuals, and it is essential to be monitored by a health professional, who will adjust the diet to the profile of each patient, considering the eating habits, nutritional status, and health conditions.

Guidelines have been emphasized for preventing diabetes and CVD in pre-diabetics, and the importance of a healthy dietary pattern containing vegetables, fruits, legumes and whole grains, as well as choosing healthy sources of protein and fresh and minimally processed foods, In addition, it is recommended to individualize eating plans with the distribution of macronutrients more consistent with personal preference and usual intake to increase the likelihood of long-term maintenance.^9^
